# Beneficial mechanisms of dimethyl fumarate in autoimmune uveitis: insights from single-cell RNA sequencing

**DOI:** 10.1186/s12974-024-03096-6

**Published:** 2024-04-29

**Authors:** Lei Zhu, He Li, Xuening Peng, Zhaohuai Li, Sichen Zhao, Dongting Wu, Jialing Chen, Si Li, Renbing Jia, Zuohong Li, Wenru Su

**Affiliations:** 1 State Key Laboratory of Ophthalmology, Zhongshan Ophthalmic Center, Guangdong Provincial Key Laboratory of Ophthalmology and Visual Science, Sun Yat-sen University, Guangzhou, 510060 China; 2 Sun Yat-sen University, Guangzhou, 510060 China; 3 Department of Ophthalmology, Shanghai Key Laboratory of Orbital Diseases and Ocular Oncology, Shanghai Ninth People’s Hospital, Shanghai JiaoTong University School of Medicine, Shanghai, 200025 China

**Keywords:** Autoimmune uveitis, Dimethyl fumarate, PIM1, CXCR4, Single-cell RNA sequencing

## Abstract

**Background:**

Dimethyl fumarate (DMF) is a fumaric acid ester that exhibits immunoregulatory and anti-inflammatory properties. However, the function of DMF in autoimmune uveitis (AU) is incompletely understood, and studies comprehensively exploring the impact of DMF on immune cells are still lacking.

**Methods:**

To explore the function of DMF in uveitis and its underlying mechanisms, we conducted single-cell RNA sequencing (scRNA-seq) on the cervical draining lymph node (CDLN) cells of normal, experimental autoimmune uveitis (EAU), and DMF-treated EAU mice. Additionally, we integrated scRNA-seq data of the retina and CDLNs to identify the potential impact of DMF on ocular immune cell infiltration. Flow cytometry was conducted to verify the potential target molecules of DMF.

**Results:**

Our study showed that DMF treatment effectively ameliorated EAU symptoms. The proportional and transcriptional alterations in each immune cell type during EAU were reversed by DMF treatment. Bioinformatics analysis in our study indicated that the enhanced expression of Pim1 and Cxcr4 in EAU was reversed by DMF treatment. Further experiments demonstrated that DMF restored the balance between effector T (Teff) /regulatory T (Treg) cells through inhibiting the pathway of PIM1-protein kinase B (AKT)-Forkhead box O1 (FOXO1). By incorporating the scRNA-seq data of the retina from EAU mice into analysis, our study identified that T cells highly expressing Pim1 and Cxcr4 were enriched in the retina. DMF repressed the ocular infiltration of Teff cells, and this effect might depend on its inhibition of PIM1 and CXCR4 expression. Additionally, our study indicated that DMF might reduce the proportion of plasma cells by inhibiting PIM1 expression in B cells.

**Conclusions:**

DMF effectively attenuated EAU symptoms. During EAU, DMF reversed the Teff/Treg cell imbalance and suppressed the ocular infiltration of Teff cells by inhibiting PIM1 and CXCR4 expression. Thus, DMF may act as a new drug option for the treatment of AU.

**Supplementary Information:**

The online version contains supplementary material available at 10.1186/s12974-024-03096-6.

## Background

Autoimmune uveitis (AU) is an ocular autoimmune-inflammatory disease that imposes a substantial risk on visual acuity [1]. Patients suffering from AU encounter significant physical and economic burdens due to the recurrence and prolonged duration of the disease [2, 3]. The primary therapeutic approach for AU involves corticosteroids and immunosuppressants; nevertheless, prolonged use of these medicines can lead to significant adverse effects both locally and systemically [4, 5]. Exploring safer and effective medicines were required to advance the prognosis of patients with AU.

Experimental autoimmune uveitis (EAU) model is extensively used for exploring AU and offers valuable insights into its pathogenesis [6]. Studies demonstrated that AU development is primarily linked to the augment of effector T (Teff) cells, particularly T helper (Th)-1 and Th17 cells, and a lack of regulatory T (Treg) cells [7–10]. Lymph nodes are immune organs where antigens are presented and immune cells are activated [11]. During the AU process, Teff cells migrate from lymph nodes to the eyes and attract inflammatory cells, ultimately resulting in retina injury and vision loss [12]. The eyes are the anatomical extension of the central nervous system (CNS) and exhibit many molecular and cellular parallels to the brain [13]. AU was also considered a kind of CNS autoimmune disease [14, 15]. Cervical draining lymph nodes are the primary draining lymph nodes of the eyes and the CNS [16, 17]. Researchers have observed efficient drainage of both macromolecules and immune cells (including T cells and dendritic cells) from the CNS to CDLNs [17]. In addition, resection or focused ultrasound treatment of CDLNs reduced the severity of experimental autoimmune encephalomyelitis, indicating the involvement of CDLNs in the pathogenesis of CNS autoimmune diseases [17, 18]. Therefore, CDLNs might be ideal for exploring immune changes of the eyes during AU. Despite advances in understanding uveitis pathogenesis, medicine options are still limited due to the high cost and long development cycle of drug targeting specific molecules. Additionally, single-molecule targeting therapy such as anti-IL-17A antibodies, showed limited efficacy in AU [19]. Therefore, repurposing existing clinical drugs may effectively supplement the treatment options for AU.

Dimethyl fumarate (DMF) is a well-characterized fumaric acid ester with immunoregulatory and anti-inflammatory effects, which has been used in the clinic [20–22]. Mechanically, studies on DMF have indicated its pleiotropic effects [23]. It has been reported that DMF reduces oxidative stress and provides neuroprotective effects [24]. DMF could also suppress NLRP3 inflammasome activation, thus exerting anti-inflammatory effects [25]. In addition, DMF inhibits the migration of immune cells across brain endothelial cells [26]. DMF has shown potential favorable effects in a range of diseases, such as hepatic ischemia-reperfusion injury [27], lymphoma [28, 29], and inflammatory bowel disorders [30]. This medicine was also approved for the treatment of multiple sclerosis and psoriasis in the clinic [31, 32]. Importantly, DMF showed long-term safety and efficacy in the treatment of these two diseases [33, 34]. Moreover, adverse events associated with DMF are typically of mild severity [35]. The broad applicability of DMF in a range of diseases, along with its safety, anti-inflammatory, and immunomodulatory properties, suggest it as a promising candidate for the treatment of AU. Thus, conducting basic experiments to explore the beneficial effects of DMF in uveitis and its underlying mechanisms is essential to support further clinical trials and potentially facilitate the application of DMF in AU treatment.

Single-cell RNA sequencing (scRNA-seq) is a powerful tool for analyzing the transcriptomes of individual cells. This tool allows for a comprehensive exploration of the impact of a drug on various immune cells within a disease, providing a detailed cellular-level understanding of the potential therapeutic mechanisms [36, 37]. In this study, scRNA-seq was utilized to fully illustrate the impact of DMF on the transcriptome of immune cells. Our results showed that DMF attenuated EAU symptoms. Genes and pathways associated with autoimmune inflammation were repressed by DMF in various immune cells. Analysis of the scRNA-seq data indicated that inhibition of PIM1 and CXCR4 might be the critical beneficial mechanism of DMF in uveitis. Subsequent experiments demonstrated that DMF enhanced the proportion of Treg cells while reducing the proportions of Th1 and Th17 cells by repressing the PIM1-AKT-FOXO1 pathway. Integrated analysis of the retinal scRNA-seq data indicated that DMF repressed the ocular infiltration of Teff cells by repressing the collaboration of PIM1 and CXCR4 in T-cell migration. In addition, DMF treatment inhibited PIM1 expression and reduced the proportion of plasma cells.

## Methods

### Animals

Female C57BL/6 mice (6–8 weeks; 18–25 g) were procured from the Animal Center located in Guangzhou. These mice were fed in a specific pathogen-free grade environment. The animal care committee at Sun Yat-Sen University approved all animal tests conducted in this study.

### EAU model development and disease grading

A 1:1 mixture containing 2 mg/ml interphotoreceptor retinoid-binding protein 1–20 (IRBP_1 − 20_, GiL Biochem, Shanghai, China) and complete Freund’s adjuvant (CFA, BD Difco, San Jose, CA, USA) with 2.5 mg/ml of *Mycobacterium tuberculosis* (BD Difco, San Jose, CA, USA) was subcutaneously injected into mice (0.2 ml per mouse) on day 0. After immunization with IRBP_1 − 20_, mice were intraperitoneally injected with 0.2 µg pertussis toxin (PTX, List Biological Laboratories, Campbell, CA, USA) dissolved in phosphate-buffered saline (0.1 ml per mouse) immediately. On day 2, PTX was again intraperitoneally injected into mice at the same dose [6]. On day 14, we conducted funduscopic examinations on the mice. The clinical scores were graded from 0 to 4 according to vasculitis and infiltration observed in the mouse retina in a blinded manner as previously reported (Details in Additional file 3) [6, 38]. Fundus photographs were taken with a fundus camera (PHOENIX, USA). Mouse eyes were collected and fixed in paraformaldehyde. Subsequently, we embedded mouse eyeballs with paraffin and sliced the eyeballs. Then, hematoxylin-eosin staining was conducted on the spliced eyeballs. The pathological scores were graded from 0 to 4 according to previously reported criteria (Details in Additional file 3) [6, 38].

### Treatment protocols

Each mouse was treated by oral gavage administration of 0.2 ml of DMF (60 mg/kg; Selleck Chemicals; USA) [39–41] or 0.2 ml of vehicle control (10% DMSO + 10%Tween 80 + 40% PEG-300 + 50%PBS) once daily for 2 weeks following immunization (start DMF administration shortly after immunization on day 0). In this treatment method, DMSO was given to mice at dosages ranging from 0.88 g/kg to 1.22 g/kg, which was lower than the reported dosage that induced toxic effects such as liver degeneration, nephritis, and death in mice [42, 43]. Another group of EAU mice were administered with DMF for 5 days (start DMF administration on day 10 after immunization) to test the therapeutic effect of DMF on established EAU. A Brief overview of the experimental design, grouping of mice, and timing of modeling and drug administration were shown (Additional file 1: Fig. S1A). For in vitro experiments, CDLN cells were isolated and administered with IRBP_1–20_ at a concentration of 20 µg/mL, either individually or in combination with DMF at a concentration of 20 µM [28, 44] for 72 h at 37 °C.

### Adoptive transfer experiment

CDLN cells were isolated from EAU mice on day 14 after immunization. These cells were stimulated with 20 µg/ml IRBP_1 − 20_ in the presence or absence of 20 μM DMF for 3 days. Following stimulation, CD4 + T cells were isolated, purified, and harvested. Subsequently, normal C57BL/6J mice were injected with CD4 + T cells via the tail vein (2 × 10^7^ cells per mouse). Each normal mouse received cells from about 5 EAU mice.

### Flow cytometry

Cells from CDLNs and the retina were harvested on day 14 after immunization. Cells were stained with live/dead dye (Thermo Fisher Scientific, Waltham, MA, USA) to identify the living cells. Subsequently, these cells were labeled with the following cell surface markers: CD4 (BioLegend, 100434), CD19 (BioLegend, 302244), Cxcr4 (BioLegend, 153805), CD138 (BioLegend, 142505), and B220 (BioLegend, 69-0452-82). For intracellular staining, cells were cultured with 1 µg/mL brefeldin A (Sigma‒Aldrich), 50 ng/mL phorbol myristate acetate (Sigma‒Aldrich, St. Louis, MO, USA), and 500 ng/mL ionomycin (Sigma‒Aldrich) for 5 h. Subsequently, the cells underwent fixation and permeabilization procedures before being stained with antibodies for various markers, including anti-IL-17A (BioLegend, 506930), anti-IFN-γ (BioLegend, 505808), anti-Foxp3 (BioLegend, 11-5773-82), anti-PIM1 (NOVUS, NBP2-67528), anti-phospho-AKT1 (BioLegend, 17-9715-42), anti-phospho-FOXO1 (Thermo Fisher Scientific, PA5-104977), and anti-rabbit IgG (H + L), F(ab’)2 fragment secondary antibody (Cell Signaling Technology, Danvers, MA, USA, 4412). Flow cytometry (BD LSRFortessa) was used for the analysis of these cells. FlowJo software was used for further results analysis.

### scRNA-seq analysis

Extracted CDLNs and retina samples were prepared as single-cell suspensions with cell viability > 85%. The single-cell suspensions of CDLN and retina were subsequently transferred into barcode scRNA-seq libraries through the Chromium Single Cell 5’ v2 Reagent (10x Genomics, 120,237) kit according to the manufacturer’s protocol. In detail, after loading the single-cell suspensions in the chromium instrument (10x Genomics), the cells were captured in single-cell gel beads. Then, reverse transcription reactions, disruption of emulsions, cDNA clean-up using DynaBeads Myone Silane Beads (Thermo Fisher Scientific), and polymerase chain reaction were performed step by step to acquire the amplified cDNA. Subsequently, the amplified cDNA was fragmented, end-repaired, A-tailed, and ligated to an index adaptor to create libraries. The created libraries were sequenced on the Illumina NovaSeq 6000 platform. We used CellRanger (v5.0.0) software for initial data processing and integration. Harmony (v0.1.0) was used to decrease batch effects. Downstream analysis was conducted using Seurat (v4.0.4). In the process of quality control, cells with fewer than 300 genes or more than 15% of the mitochondrial genes were filtered out.

#### Differentially expressed gene (DEG) and Gene Ontology (GO) analysis

DEG analysis in different cell types between various groups was conducted through the “FindMarkers” function using default parameters in Seurat packages. DEGs were defined as genes with adjusted P values < 0.05 and | Log2 (fold change) |>0.25 (a list of DEGs was in Additional file 2). The Metascape online web tool (www.metascape.org) was used for GO analysis. This web tool utilized input DEGs to generate GO terms. Among the top 50 GO terms, the GO terms related to disease were visualized via the ggplot2 R package.

#### Trajectory analysis

The Monocle2 R package (V4.0.0) was used to perform trajectory analysis of CD4 + T-cell subsets and B-cell subsets. Equal numbers of cells were randomly selected from each group for trajectory analysis. Genes highly expressed in each cell subset generated using the “FindallMarkers” function in Seurat were set as ordering genes.

#### Intercellular communication

The scRNA-seq data of the retina from EAU mice was used for intercellular communication analysis. CellChat [45] R package (v4.0.0) was utilized to construct the intercellular communication network and predict the strength of various signals between different cells by assessing the expression of ligand-receptor pairs within cells.

### Statistical analysis

Data analysis and presentation were conducted using GraphPad Prism (version 8.0.2). The results were shown as the mean ± standard deviation (SD). The clinical and pathological scores of fundi were analyzed through the Mann-Whitney U test or the Kruskal Wallis Test. Shapiro-Wilk test was used to determine normal distribution. In case of normal distribution, significance was determined through unpaired two-tailed Student’s t-tests or one-way analysis of variance (ANOVA). A significance threshold of *P* < 0.05 was applied to determine statistical significance in the study.

## Results

### DMF ameliorated the symptoms and reversed transcriptional alterations of EAU

**Fig. 1:**
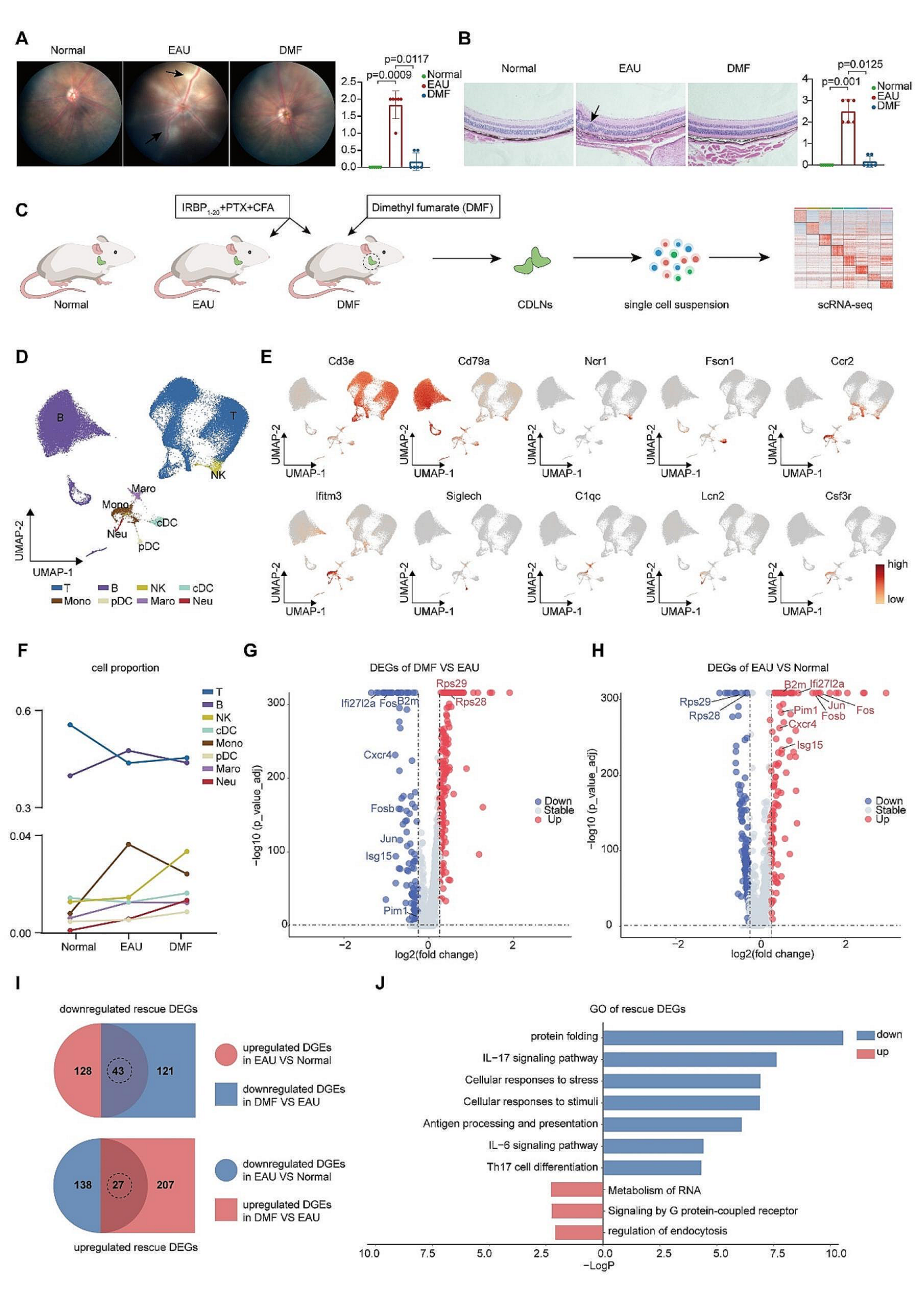
**scRNA-seq analysis of immune cells during EAU and post-DMF administration**. A. Representative fundus images and clinical scores of normal, EAU, and DMF-treated EAU mice. Arrows indicate inflammatory exudation and vascular deformation. Each group contained six mice. The values represent the mean ± SD. Significance was determined using Kruskal Wallis Test. B. Representative fundus hematoxylin and eosin staining plots and pathological scores of normal, EAU, and DMF-treated EAU mice. Arrows indicate retinal folding and inflammatory cell infiltration. Scale bars, 20 mm. Each group contained six mice. The values represent the mean ± SD. Significance was determined using Kruskal Wallis Test. C. Scheme of the overall study design. IRBP_1 − 20_: interphotoreceptor retinoid-binding protein 1–20; PTX: pertussis toxin; CFA: complete Freund’s adjuvant. D. UMAP plots of immune cell clusters from the CDLNs of all the mouse groups. E. UMAP plots of canonical markers of immune cell clusters from all the mouse groups. F. Line charts showing the percentages of major immune cell types in normal, EAU, and DMF-treated EAU mice. G-H. Volcano plots showing upregulated and downregulated DEGs of all immune cell types in the DMF-treated EAU/EAU (G) and EAU/Normal (H) comparison groups. The red and blue dots indicate upregulated and downregulated DEGs, respectively. I. Venn diagrams showing the numbers of rescue DEGs of all immune cells. The overlapping parts indicate the numbers of downregulated rescue DEGs (top) and upregulated rescue DEGs (bottom). J. Bar plot showing GO terms enriched by downregulated or upregulated rescue DEGs of all immune cells

An EAU model was established using IRBP_1 − 20_, PTX, and CFA as previously reported [6]. After immunization, the EAU mice were daily received oral administration of DMF for 14 days. DMF administration efficiently ameliorated EAU symptoms and reduced the clinical and pathological scores (Fig. 1A and B). Additionally, to test the therapeutic effect of DMF on established EAU, we administered established EAU mice (day 10 after immunization) with DMF for 5 days. We found that the therapeutic effect still existed (Additional file 1: Fig. S1B). In the following experiments, we started DMF administration on the first day of modeling in the DMF group.

To identify the genomic variations associated with DMF administration, scRNA-seq was performed on isolated CDLN cells derived from normal mice, EAU mice, and EAU mice administered with DMF (Fig. 1C). After initial processing, including quality filtering, integration, and batch effect correction, the cells were divided into 8 distinct clusters. Uniform manifold approximation and projection (UMAP) was used for visualization (Fig. 1D). The cell clusters were annotated via their expression of marker genes (Fig. 1D and E, Additional file 1: Fig. S1C). 8 classical immune cell types, namely, T cells, B cells, natural killer cells (NKs), conventional dendritic cells (cDCs), monocytes (Monos), plasmacytoid dendritic cells (pDCs), macrophages (Macros), and neutrophils (Neus), were identified. We then explored the proportional alterations of immune cells post-DMF administration. The proportional changes during EAU were partly reversed by DMF treatment in several immune cell types, including T cells, B cells, monocytes, pDCs, and macrophages (Fig. 1F). To explore the overall effect of DMF on the transcriptome of EAU mice, we performed DEG analysis of all immune cells between the DMF and EAU groups as well as between the EAU and normal groups (Fig. 1G and H). DMF reduced the expression of genes related to AP-1 (*Fos, Fosb, and Jun*), antigen presentation (*B2m*), interferon signaling (*Ifi27l2a and Isg15*), lymphocyte differentiation (*Pim1*), and cell migration (*Cxcr4*) (Fig. 1G). These genes were included in the DEGs which were upregulated during EAU compared to the normal condition (Fig. 1H). Meanwhile, DMF treatment upregulated two genes involved in RNA metabolism, *Rps28* and *Rps29*, which were downregulated during EAU (Fig. 1G and H). These results indicated that DMF partially reversed the EAU-induced transcriptional changes. To investigate the specific beneficial mechanisms of DMF on EAU, we further identified “rescue DEGs” to better elucidate the EAU-induced transcriptional alterations that were reversed following DMF treatment (Fig. 1I). The genes upregulated in EAU and downregulated in the DMF group were identified as downregulated rescue DEGs. Similarly, the genes downregulated in EAU and upregulated in the DMF group were identified as upregulated rescue DEGs (Fig. 1I). Subsequently, a GO analysis was performed to characterize the biological significance of these rescue genes. Pathways related to IL-17 signaling, antigen processing and presentation, IL-6 signaling, and Th17 cell differentiation were enriched by the downregulated rescue DEGs (Fig. 1J). Meanwhile, pathways related to RNA metabolism, G protein-coupled receptor signaling, and endocytosis were enriched by the upregulated rescue DEGs (Fig. 1J). These results indicated that DMF partly reversed EAU-induced transcriptional alterations. We also conducted DEG analysis between the DMF group and the normal group (Additional file 1: Fig. S1D). We observed that the expression of *Fos*, *Ifi27l2a*, *Isg15*, and *Cxcr4* was lower in DMF group compared to normal group (Additional file 1: Fig. S1D). In addition, the expression of genes related to the function of macrophages and neutrophils (*Ccl5*, *Ctsb*, and *Grn*) was higher in the DMF group compared to the normal group. GO analysis was conducted to annotate these DEGs. The downregulated DEGs in the DMF group compared to the normal group were enriched in pathways related to electron transport chain and ATP process, while the upregulated DEGs were enriched in pathways related to neutrophil degranulation and innate immune response (Additional file 1: Fig. S1E).

Overall, DMF treatment efficiently ameliorated EAU symptoms and partly reversed EAU-induced transcriptional alterations.

### DMF partly reversed the transcriptional alterations among the major immune cells

**Fig. 2:**
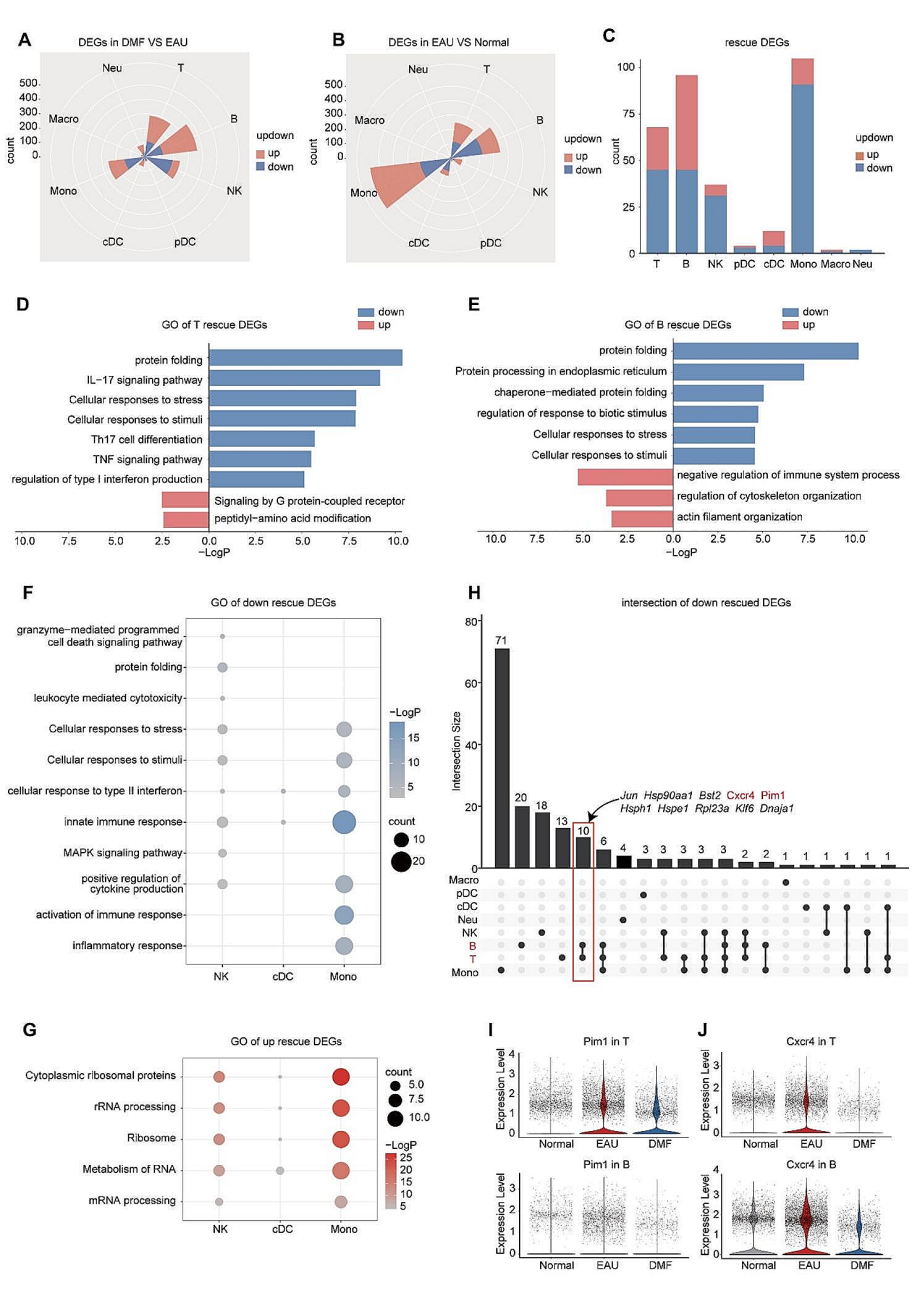
**scRNA-seq analysis of the rescue DEGs in the major immune cell types**. A-B. Rose diagrams showing the numbers of upregulated and downregulated DEGs of the major immune cell types in the DMF-treated EAU/EAU (A) and EAU/Normal comparison groups (B). C. Bar plot showing the numbers of upregulated and downregulated rescue DEGs of the major immune cell types. D-E. Bar plot showing GO terms enriched by downregulated or upregulated rescue DEGs in T cells (D) and B cells (E). F-G. Dot plot showing GO terms enriched in downregulated (F) and upregulated (G) rescue DEGs in NKs, cDCs, and monocytes. H. UpSet plot showing the downregulated rescue DEGs in eight types of cells. Genes in both T and B cells are labeled at the top. I-J. Violin plots showing the expression of Pim1 (I) and Cxcr4 (J) in T and B cells from normal, EAU, and DMF-treated EAU mice

Next, we examined the influence of DMF on transcriptomic profiles of the major immune cell types. DEG analysis was conducted, and rescue DEGs were identified for each major immune cell type (Fig. 2A-C). Most rescue DEGs were found in T cells, B cells, and monocytes (Fig. 2C). Subsequently, GO analysis was performed. In T cells, their downregulated rescue DEGs were involved in pathways associated with IL-17 signaling, Th17 cell differentiation, TNF signaling, and interferon production, whereas the upregulated ones were involved in pathways associated with G protein-coupled receptor signaling and peptidyl-amino acid modification (Fig. 2D). For B cells, pathways associated with cellular responses to stress and stimuli were down-rescued, whereas pathways associated with the negative regulation of immune system processes, regulation of cytoskeleton organization, and actin filament organization were up-rescued (Fig. 2E). For NKs, cDCs, and monocytes, pathways associated with the cellular response to interferon and the innate immune response were down-rescued, whereas pathways associated with RNA processing and metabolism were up-rescued (Fig. 2F and G). Additionally, the pathway related to cytotoxicity and the pathway associated with inflammatory response was down-rescued in NKs and monocytes, respectively (Fig. 2G). The number of rescue DEGs of pDCs, macrophages, and neutrophils was limited; therefore, we performed GO analysis on the downregulated or upregulated DEGs in the DMF group compared to the EAU group. In pDCs and macrophages, pathways associated with cellular responses to stress and stimuli, and antigen processing and presentation were downregulated, whereas pathways associated with RNA processing were upregulated after DMF treatment (Additional file 1: Fig. S1F and G). In neutrophils, the downregulated DEGs post-DMF administration were associated with the cytokine signaling-related pathway. The upregulated DEGs of neutrophils were involved in pathways associated with ribonucleoside metabolic and biosynthetic processes (Additional file 1: Fig. S1F and G). These results indicated that DMF might exert its effects through downregulation of several autoimmune- and inflammation-related genes that were upregulated in EAU.

To further identify the critical mediators of effects of DMF, we generated an UpSet plot to visualize the common and specific downregulated rescue DEGs among the major immune cell types (Fig. 2H). We observed that 10 genes (*Jun*, *Hsp90aa1*, *Bst2*, *Cxcr4*, *Pim1*, *Hsph1*, *Hspe1*, *Rpl23a*, *Klf6*, and *Dnaja1*) were uniquely down-rescued in T and B cells (Fig. 2H). Among these genes, *Hsp90aa1*, *Hsph1*, *Hspe1*, and *Dnaja1* encode molecular chaperones and cochaperones for protein folding [46, 47]. *Rpl23a* encodes a ribosomal protein. Studies on *Jun*, *Bst2*, and *Klf6* have indicated the involvement of these molecules in a variety of tumors [48–51]. *Pim1* has been reported to regulate the differentiation of T and B cells [52, 53]. *Pim1* also promotes Cxcr4 cell surface expression, thus regulating cell migration [54]. Intriguingly, *Pim1* and *Cxcr4*, two genes actively involved in lymphocyte differentiation and migration [52–54] were down-rescued in T and B cells by DMF (Fig. 2I and J). These results indicated that DMF might exert its effect on uveitis by regulating Pim1 and Cxcr4 expression.

### DMF reversed the proportional and transcriptional alterations of T-cell subsets during EAU

**Fig. 3:**
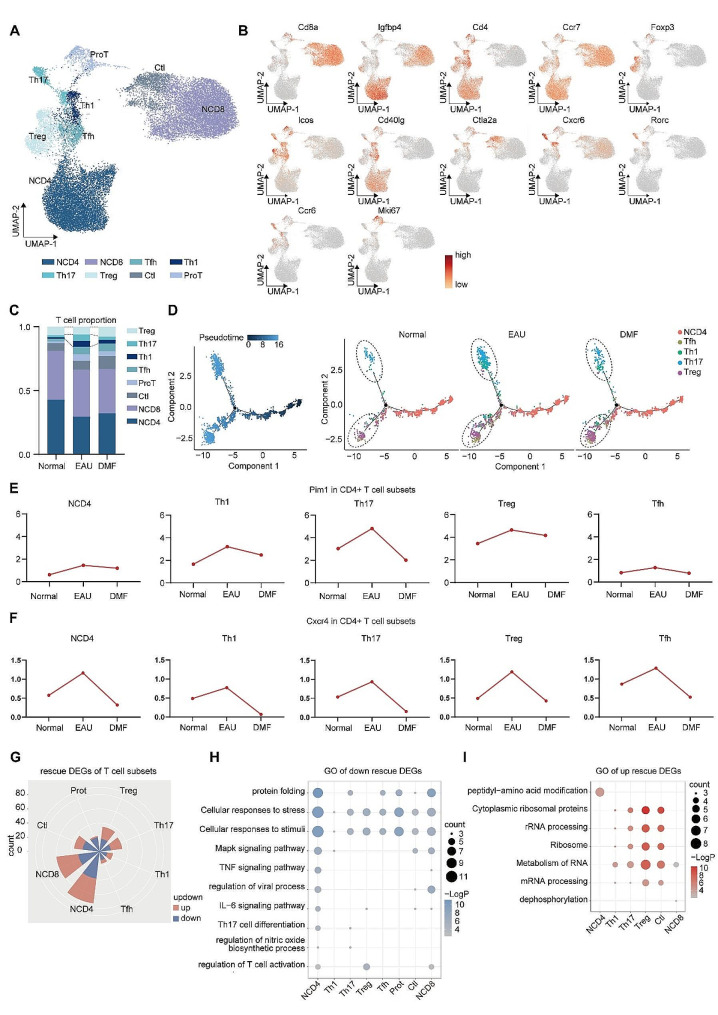
**DMF partly reversed the proportional and transcriptional alterations in T-cell subsets during EAU**. A. UMAP plots of T-cell subsets from the CDLNs of all the mouse groups. B. UMAP plots of canonical markers of T-cell subsets from all the mouse groups. C. Bar plots showing the percentages of T-cell subsets among CDLNs from normal, EAU, and DMF-treated EAU mice. D. Pseudotime trajectory analysis of CD4 + T cells. Cells are colored according to pseudotime (left) or cell type (right). E-F. Line plots showing the expression of Pim1 (E) and Cxcr4 (F) in each CD4 + T-cell subset. G. Rose diagram showing the numbers of upregulated and downregulated rescue DEGs in each T-cell subset. H. Dot plot showing GO terms enriched by downregulated rescue DEGs in NCD4, Tfh, Th1, Th17, Treg, Prot, Ctl, and NCD8 cells. I. Dot plot showing GO terms enriched by upregulated rescue DEGs in NCD4, Th1, Th17, Treg, and NCD8 cells

Considering the critical role of T cells in EAU pathogenesis, we reclustered T cells and identified 8 cell clusters, including naïve CD4 + T (NCD4) cells, naïve CD8 + T (NCD8) cells, T follicular helper (Tfh) cells, Th1 cells, Th17 cells, Treg cells, cytotoxic T lymphocytes (Ctl), and proliferative T (Prot) cells (Fig. 3A and B, Additional file 1: Fig. S2A). CD4 + T cells are actively involved in EAU development. In our data, DMF counteracted the elevated frequencies of Th1 and Th17 cells during EAU, while concurrently promoting an expansion in the Treg cell population. In addition, the proportion of effector CD8 + T cells, namely Ctl cells, was not rescued by DMF treatment (Fig. 3C). Considering the altered proportions in CD4 + T cell subsets, we conducted trajectory analysis to explore the effect of DMF on CD4 + T cell differentiation (Fig. 3D). We observed an increased trend from NCD4 cells to Th1 and Th17 cells during EAU, which was reversed post-DMF administration (Fig. 3D). Meanwhile, the trend toward Treg cells also enhanced by DMF treatment (Fig. 3D). Subsequently, we assessed the expression levels of Pim1 and Cxcr4 within individual CD4 + T cell subpopulations. The expression of Pim1 was significantly upregulated during EAU in Th1 cells, Th17 cells, and Treg cells, and down-rescued by DMF (Fig. 3E). Considering the role of Pim1 in regulating T cell differentiation, DMF may reverse the Teff/Treg cell imbalance by regulating Pim1 during uveitis. Moreover, the expression of Cxcr4 was upregulated in EAU and repressed by DMF treatment in all CD4 + T-cell subpopulations (Fig. 3F).

To investigate the transcriptional alterations within individual T-cell subpopulations, GO analysis was performed on the rescue DEGs within each T-cell subpopulation. Most rescue DEGs were detected in the NCD4 and NCD8 cells (Fig. 3G). Pathways associated with protein folding, and cellular responses to stress and stimuli were down-rescued in most T cell subpopulations (Fig. 3H). The pathway related to Th17 cell differentiation was down-rescued in NCD4 and Th17 cells (Fig. 3H). In addition, the pathways associated with TNF signaling and T-cell activation were down-rescued in NCD4 and NCD8 cells (Fig. 3H). For GO analysis of upregulated rescue genes, we observed that pathways related to RNA processing and metabolism were up-rescued in most T cell subsets and the pathway annotated as “peptidyl-amino acid modification” was up-rescued in NCD4 cells (Fig. 3I). The amount of upregulated rescue DEGs of Tfh and Prot cells was limited. Thus, we conducted GO analysis on their upregulated DEGs in the DMF group compared with the EAU group and identified enriched pathways related to peptidyl-amino acid modification and RNA processing (Additional file 1: Fig. S2B and C). Collectively, the altered expression of Pim1 and Cxcr4 in CD4 + T cell subpopulations during EAU and post-DMF administration indicated the potential involvement of these two molecules in the beneficial effects of DMF administration. In addition, the results of GO analysis demonstrated that DMF reversed the transcriptional alterations of T-cell subsets during EAU.

### DMF reversed the Teff/Treg imbalance by inhibiting the PIM1-AKT-FOXO1 pathway

**Fig. 4:**
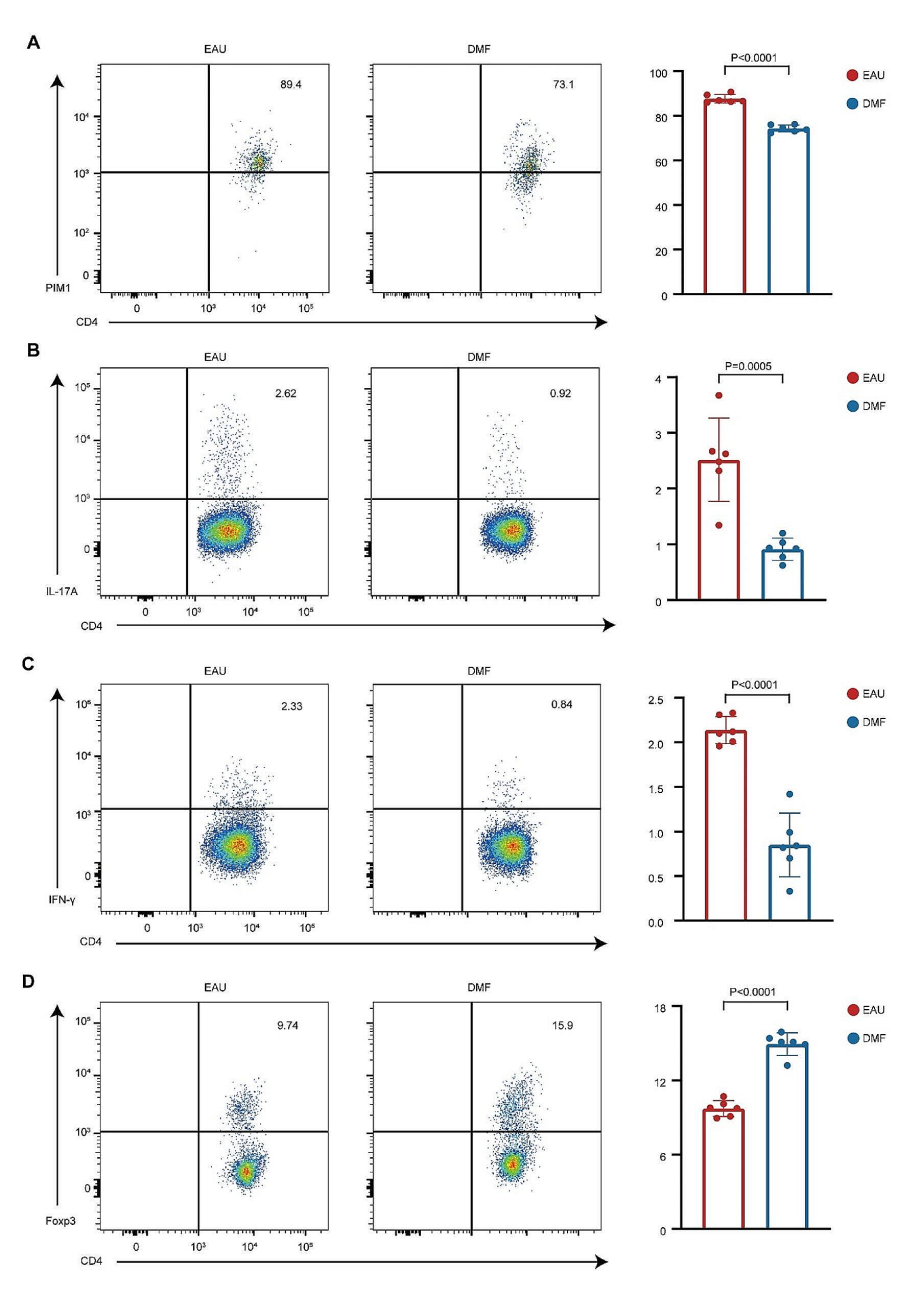
**DMF reduced PIM1 expression in CD4 + T cells and reversed the Teff/Treg imbalance**. A-D. The proportions of PIM1 + cells (A), Th17 cells (B), Th1 cells (C), and Treg cells (D) among CD4 + T cells from CDLNs of EAU mice and DMF-treated EAU mice were measured via flow cytometry after immunization on day 14. Each group contained six mice. The data are expressed as the mean ± SD. Significance was determined using unpaired two-tailed Student’s t-test

Augment Teff (Th1 and Th17) cells and insufficient Treg cells, namely, the Teff/Treg imbalance, are critical pathogenic factors in AU [55, 56]. Our above analysis indicated that DMF might regulate CD4 + T-cell differentiation by regulating PIM1. To verify our hypothesis, flow cytometry was performed on CDLN cells isolated from EAU group or DMF group. DMF treatment reduced PIM1 expression in CD4 + T cells during EAU (Fig. 4A). Additionally, the proportions of Th17 cells (CD4 + IL17A + T cells) and Th1 cells (CD4 + IFN-γ + T cells) were significantly decreased, whereas the proportion of Treg cells (CD4 + Foxp3 + T cells) was significantly enhanced in the DMF group compared to the EAU group (Fig. 4B-D). Therefore, DMF might reverse the imbalance of Teff/Treg by inhibition of PIM1 expression.

**Fig. 5:**
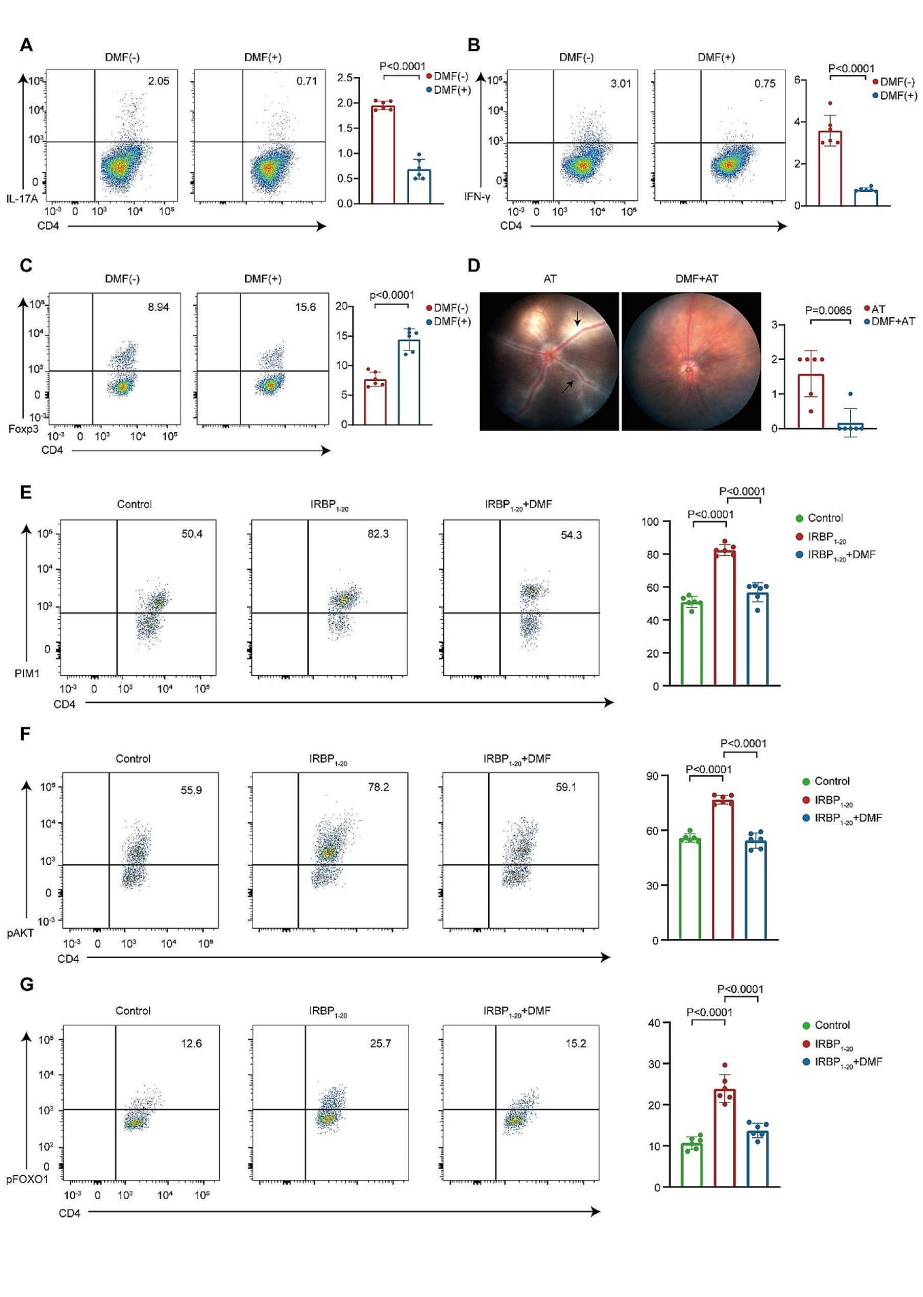
**DMF reversed the Teff/Treg imbalance by regulating PIM1-AKT-FOXO1**. A-C. CDLN cells from EAU mice were cultured with IRBP1-20 or IRBP1-20 plus DMF. Flow cytometry was performed to determine the proportions of Th17 cells (A), Th1 cells (B), and Treg cells (C). Each group contained six mice. The data are expressed as the mean ± SD. Significance was determined using an unpaired two-tailed Student’s t-test. D. Representative fundus images and clinical scores of mice transferred with CD4 + T cells cultured with IRBP1-20 or IRBP1-20 plus DMF after 14 days of modeling. White arrowheads indicate inflammatory exudation and vascular deformation. Each group contained six mice. The data are expressed as the mean ± SD. Significance was determined using Mann-Whitney U test. AT: Adoptive transfer. E-G. CDLN cells from the EAU group were cultured with IRBP1-20 alone or with IRBP1-20 plus DMF for 72 h. Flow cytometry showing the proportions of PIM1 + cells (E), pAKT + cells (F), and pFOXO1 + cells (G) among the CD4 + T cells. The data are presented as the means ± SD from six independent experiments. Significance was determined using one-way ANOVA

To further identify the role of DMF in CD4 + T cell subsets, in vitro experiments were also performed. We isolated CDLN cells of EAU mice and cocultured these cells with IRBP_1 − 20_ or IRBP_1 − 20_ plus DMF. The treatment with DMF resulted in a reduction in the proportions of Th17 and Th1 cells, along with an augmentation in Treg cell proportion (Fig. 5A-C). Meanwhile, upon transferring these CD4 + T cells into naive mice, we noted that DMF treatment diminished the capacity of autoreactive CD4 + T cells to instigate EAU (Fig. 5D).

Next, we delved deeper into elucidating the regulatory mechanisms by which DMF impacts the Teff/Treg cell balance. PIM1 is able to modulate CD4 + T-cell differentiation through phosphorylation of the AKT-FOXO1 pathway [53]. The transcriptional factor, FOXO1, promotes Treg differentiation via induction of FOXP3 expression but represses Th1 and Th17 differentiation via inhibition of T-bet and RORγ [57–60]. PIM1 enhances AKT phosphorylation and activates its kinase activity [53]. FOXO1 phosphorylated by AKT translocates from the nucleus to the cytosol, resulting in loss of its regulatory activity [57, 61]. Thus, we performed flow cytometry to determine the influence of DMF on the PIM1-AKT-FOXO1 pathway. Upon stimulation with IRBP_1 − 20_, the expression of PIM1 and the phosphorylation levels of AKT and FOXO1 were enhanced in CD4 + T cells. These alterations induced by IRBP_1 − 20_ were reversed by DMF treatment (Fig. 5E-G). Thus, DMF might reverse the Teff/Treg imbalance by regulating the PIM1-AKT-FOXO1 pathway.

In addition, we orally administered DMF to normal mice for 14 days to investigate the potential effects of DMF on normal mice. We observed that normal mice administered with DMF didn’t show any retina lesions in the funduscopic examinations and the eyeball pathological sections on day 14 after treatment initiation (Additional file 1: Fig. S3A and B). Furthermore, flow cytometry analysis showed no significant differences in the proportions of Th1, Th17, and Treg cells among CDLN cells between normal mice and those treated with DMF (Additional file 1: Fig. S3C-E). These results indicated that DMF administration had no significant effect on the proportion of Th1, Th17, and Treg cells in normal mice.

### DMF reversed the proportional and transcriptional alterations in B-cell subsets during EAU

Currently, the role of B cells has received increasing attention in immune-related disorders. Next, we studied the effect of DMF on B cells. We reclustered B cells and identified 3 cell clusters, namely, naïve B cells (NBCs), germinal center B cells (GCs), and plasma cells (PCs), according to their expression of classical markers (Additional file 1: Fig. S4A- C). During EAU, there was an increase in the proportions of PCs and GCs, which were reversed by DMF treatment (Additional file 1: Fig. S4D). To identify the impact of DMF on the differentiation of B cells, we conducted trajectory analysis on B-cell subsets. The trend toward GCs and PCs was increased during EAU and reduced post-DMF administration (Additional file 1: Fig. S4E). Pim1 has been reported to support B-cell differentiation [53]. Next, we evaluated Pim1 expression in B-cell subsets. Among all B-cell subsets, the expression of Pim1 increased in EAU and was repressed by DMF administration (Additional file 1: Fig. S4F). Further, flow cytometry was conducted to validate the effect of DMF on B cells. DMF treatment reduced PIM1 expression and PC proportion among B cells (Additional file 1: Fig. S4G and H). Thus, DMF might reduce the proportion of PC by repressing PIM1 expression. We also evaluated Cxcr4 expression. DMF reversed the enhanced Cxcr4 expression in NBCs during EAU but did not reverse the altered Cxcr4 expression in GCs and PCs (Additional file 1: Fig. S4I).

Subsequently, the influence of DMF on the transcriptomes of B-cell subpopulations was explored. DEG analysis was conducted. The greatest number of rescued DEGs were detected in NBCs (Additional file 1: Fig. S4J). GO analysis of the downregulated rescue DEGs revealed that in NBCs and PCs, pathways associated with cellular responses to stress and stimuli and regulation of lymphocyte activation were down-rescued (Additional file 1: Fig. S4K). The amount of downregulated rescue DEGs of GCs was limited. Thus, we performed GO analysis on the downregulated DEGs of GCs in DMF group compared to EAU group and identified enriched pathways related to translation and peptide biosynthetic process (Additional file 1: Fig. S4L). Meanwhile, GO analysis of the upregulated rescue DEGs showed that the pathway related to RNA metabolism was up-rescued in both NBCs and GCs (Additional file 1: Fig. S4M). There were no upregulated rescue DEGs in PCs. GO analysis of the upregulated DEGs of PCs in DMF group compared to EAU group revealed enrichment in the pathway related to intracellular signaling by second messengers (Additional file 1: Fig. S4N). Collectively, DMF reversed the proportional and transcriptional alterations in B-cell subsets during EAU.

### DMF decreased T cell ocular infiltration

**Fig. 6:**
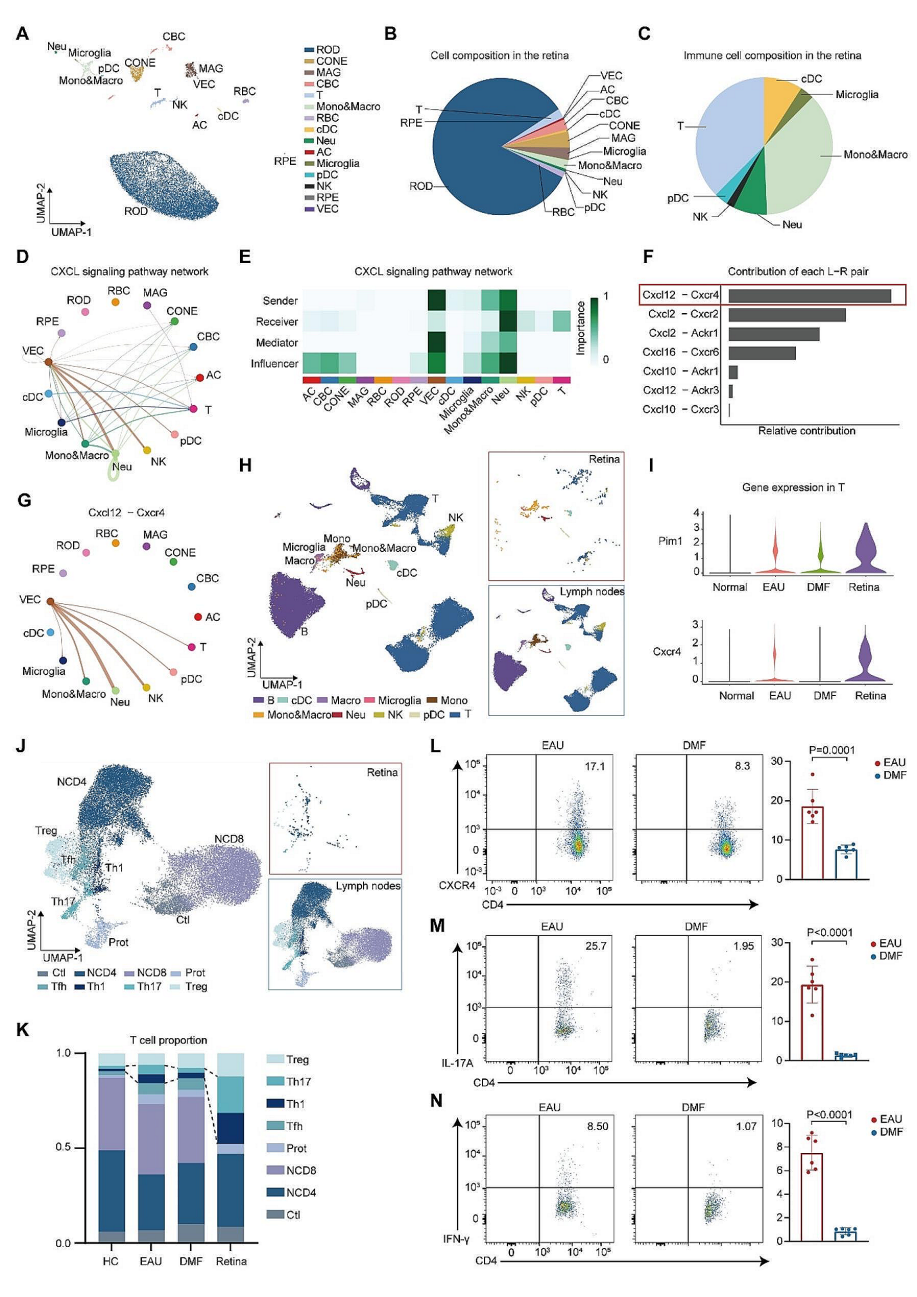
**scRNA-seq analysis of the retina from EAU mice**. A. UMAP plot of retina cells from EAU mice. B-C. Pie charts showing the cell composition (B) and immune cell composition (C) of the retina. D. The inferred CXCL signaling network between different cell clusters. The edge width represents the communication probability. E. Heatmap of the CXCL signaling network displaying the relative importance of each cell group. F. Relative contribution of each ligand-receptor pair in the communication network of the CXCL signaling pathway. G. The inferred Cxcl12-Cxcr4 intercellular network between different cell clusters. The edge width represents the communication probability. H. UMAP plots of integrated immune cells from the retina of EAU mice and CDLNs of all the mouse groups. I. Violin plots showing the expression of Pim1 and Cxcr4 in T cells from the CDLNs of normal, EAU, and DMF-treated EAU mice as well as from the retina of EAU mice. J. UMAP plots of integrated T cells from the retina of EAU mice and CDLNs of all the mouse groups. K. Bar plots showing the percentages of T-cell subsets from CDLNs of normal, EAU, and DMF-treated EAU mice as well as from the retina of EAU mice. L. The proportions of Cxcr4 + cells among CD4 + T cells from CDLNs of EAU mice and DMF-treated EAU mice were measured via flow cytometry after immunization on day 14. Each group contained six mice. The data are expressed as the mean ± SD. Significance was determined using unpaired two-tailed Student’s t-test. M-N. The proportions of Th17 cells (M) and Th1 cells (N) among ocularly infiltrated CD4 + T cells were measured by flow cytometry after immunization on day 14. Each group contained six mice. The data are expressed as the mean ± SD. Significance was determined using unpaired two-tailed Student’s t test

Uveitis is characterized by the infiltration of T cells and other leukocytes into the eyes [12]. To determine the impact of DMF on ocular infiltrating immune cells and the underlying mechanism involved, we integrated scRNA-seq data from retina samples derived from EAU mice into our present study. Retinal cells were clustered and visualized via UAMP. Fifteen cell clusters were identified, namely, rod cells (RODs), cone cells (CONEs), macroglia (MAGs), cone bipolar cells (CBCs), T cells, monocytes and macrophages (Mono&Macro), rod bipolar cells (RBCs), cDCs, neutrophils, amacrine cells (ACs), microglia, pDCs, NK cells, retinal pigmented epithelial cells (RPEs), and vascular endothelial cells (VECs). Retinal monocytes and macrophages were mixed into a cluster, namely, Mono&Macro (Fig. 6A, Additional file 1: Fig. S5A and B). Besides retinal intrinsic cells, retinal cells in our data contained quite a few infiltrated immune cells (Fig. 6B). Among the retinal immune cells, T cells, Mono&Macro accounted for the greatest percentage of cells (Fig. 6C). We then conducted CellChat to infer the intercellular communication network among retinal cells during EAU (Additional file 1: Fig. S5C). Our above results indicated decreased expression of Cxcr4 in T cells post-DMF administration. Thus, we identified the CXCL signaling pathway network among retina cells to identify the role of Cxcr4 in ocular immune cell infiltration (Fig. 6D). VECs were the major senders of CXCL signaling, whereas neutrophils and T cells were the major receivers (Fig. 6E). Contribution prediction indicated that Cxcl12-Cxcr4 was the most significant ligand-receptor pair among CXCL signaling (Fig. 6F). VECs were the major source of Cxcl12 and acted on Cxcr4 expressed on microglia and infiltrated immune cells (Fig. 6G). Thus, these results indicated that the ligand-receptor pair Cxcl12-Cxcr4 actively engaged in immune cell infiltration during EAU, which might be reversed by DMF.

Peripheral T cells infiltrating into the eyes is an important pathogenic factor for uveitis [12]. Our above data showed that DMF reduced the expression of Cxcr4 and Pim1. Cxcr4 was actively involved in cell migration [62]. Pim1 promotes the cell surface expression of Cxcr4 thus regulating CXCR4-dependent cell migration [54]. To explore the impact of DMF on ocular T cell infiltration during uveitis, we integrated retinal immune cells of EAU mice with CDLN cells from each group and observed that T cells in the retina of EAU mice expressed the highest level of Cxcr4 and Pim1 (Fig. 6H and I). In addition, we reclustered T cells from CDLNs and the retina, and found that the proportions of Th1 and Th17 cells in the retina during EAU were much higher than those in CDLNs of each group (Fig. 6J and K). Subsequently, flow cytometry was utilized to exam the function of DMF on Cxcr4 expression and ocular T-cell infiltration. DMF reduced CXCR4 expression in CD4 + T cells in CDLNs (Fig. 6L). The proportions of ocular infiltrating Th17 and Th1 cells were significantly lower in the DMF group than in the EAU group (Fig. 6M and N). Thus, DMF treatment could inhibit ocular infiltration of Teff cells in uveitis and this effect might depend on its inhibition of PIM1 and CXCR4 expression.

## Discussion

In the current study, we elucidated the effects of DMF in EAU and its underlying mechanisms. We established a post-DMF administration transcriptional atlas to depict the regulatory effect of DMF on each immune cell type during uveitis. Our results showed that DMF partly reversed the transcriptional alterations observed during uveitis. Downregulated rescue DEGs, namely, the DEGs upregulated in EAU and downregulated after DMF treatment, showed enrichment in pathways related to autoimmune inflammation. Subsequent studies demonstrated that PIM1 and CXCR4 were involved in the beneficial mechanism of DMF treatment. We identified that DMF reduced the proportions of pathogenic cell types (including Th1 and Th17 cells), while concurrently increasing the frequency of Treg cells via repression of the PIM1-AKT-FOXO1 pathway. Furthermore, our study validated that DMF might inhibit ocular infiltration of Teff cells through suppressing the collaboration of PIM1 and CXCR4. Additionally. DMF also reduced the PIM1 expression and the proportion of PCs. Therefore, our study indicated that PIM1 and CXCR4 might be critical mediators of the effects of DMF in uveitis.

DMF has been reported to have beneficial effects in treating a wide range of diseases and has been approved for treating multiple sclerosis and psoriasis in the clinic [31, 32]. However, there is limited research on the impact of DMF on uveitis, and we could only retrieve two relevant articles [63, 64]. A study published in 2007 explored the effect of fumaric acid esters in 4 patients with uveitis (3 patients with intermediate uveitis and 1 patient with Birdshot chorioretinopathy) [63]. This study observed that fumaric acid esters were able to reduce the dosage of corticosteroids, reduce cystoid macular edema, and improve vision [63]. While this study was innovative, it was preliminary as it only included 4 patients. In 2021, Labsi et al. reported the beneficial effects of DMF on EAU rats [64]. In that study, DMF reduced the symptoms of EAU and decreased the level of nitric oxide and TNF-α in the plasma of EAU rats [64]. DMF also decreased iNOS, CD68, and CD20 expression as well as increased the CD25 expression in retina tissues of EAU rats [64]. However, this study did not explore the regulatory mechanisms of DMF in the key pathogenic process of uveitis, namely Teff/Treg imbalance and T cell ocular infiltration. Consistent with previous studies, we found that DMF effectively ameliorated EAU symptoms in mice and increased the proportion of Treg cells (CD25 + cells). In addition, our study constructed a post-DMF administration immune cell atlas using scRNA-seq, through which we identified novel mechanisms under the beneficial effects of DMF in uveitis. In our data, genes enriched in pathways related to Th17 cell response, antigen processing and presentation, as well as inflammation were repressed by DMF. These pathways were closely related to autoimmune inflammation [65, 66]. Importantly, we identified Cxcr4 and Pim1, two molecules involved in immune cell differentiation and migration [53, 67, 68], were down-rescued by DMF treatment in T and B cells. This result indicated the involvement of these two molecules in the beneficial mechanism of DMF.

CD4 + T cells are important components of the autoimmune immune response. Expanded Teff cells, as well as insufficient Treg cells, have been implicated in the pathogenesis of various autoimmune disorders, such as AU [55, 69]. DMF has been reported to reduce the abundance of Th1 and Th17 cells while enhancing that of Treg cells during psoriasis and multiple sclerosis [70, 71]. Previous study indicated that T cells from peripheral blood co-cultured with DMF exhibited inhibition of activation and proliferation in response to stimulation due to the oxidative effects of DMF, while Treg cells have higher ability to resist oxidative stress [70]. Another study revealed that DMF induced apoptosis of T cells by targeting NF-κB in T cell lymphoma [72]. However, the mechanism of DMF in regulating Teff/Treg balance during uveitis has not been reported. Our study identified a new mechanism of DMF to regulate T cells, namely by modulating PIM1 expression. Previous studies revealed that PIM1-AKT-FOXO1 signaling promotes an imbalance of Teff/Treg cells [53]. In the nucleus, FOXO1 promotes FOXP3 expression to induce Treg cell differentiation, while inhibiting T-bet and RORγ expression to represses the differentiation of Th1 and Th17 cells [57–60]. PIM1 activates the kinase activity of AKT, thus promoting the phosphorylation of its downstream molecule FOXO1 [53]. Phosphorylated FOXO1 translocates into the cytoplasm, and loses its ability in Treg cell promotion and Teff cell inhibition [57]. Our study revealed that in CD4 + T cells, IRBP_1 − 20_ stimulation enhanced the PIM1 expression and the phosphorylation level of AKT-FOXO1. These alterations in PIM1-AKT-FOXO1 signaling were reversed by DMF treatment. Thus, DMF may reverse the Teff/Treg imbalance during EAU via inhibiting the PIM1-AKT-FOXO1 pathway. Increasing evidence has also indicated the involvement of autoantibodies produced by PCs in uveitis development [73, 74]. PIM1 was able to promote PC differentiation [53]. Our study demonstrated that DMF treatment reduced the PIM1 expression in B cells and the proportion of PCs, indicating that DMF reduced the proportion of PC via repression of Pim1 expression.

Immune cell ocular infiltration is one of the characteristics of uveitis [12]. As shown by our data, T cells account for a large portion of the retina-infiltrating immune cells during EAU. Furthermore, there was a greater presence of Th1 and Th17 cells in the retina than in CDLNs, indicating active migration of Teff cells from peripheral lymph nodes into the retina. In active multiple sclerosis, CXCR4 + leukocytes are trafficked to the brain lesions to cause tissue damage by CXCL12 secreted by endothelial cells [75]. CXCR4 was also reported to be related to EAU pathogenesis [76]. The intercellular communication analysis in our study among the retina cells predicted that Cxcl12-Cxcr4 was a significant ligand-receptor pair that was mainly sent by VECs and acted on immune cells, indicating the involvement of CXCR4 in ocular infiltration of immune cells during uveitis. PIM1 has been reported to promote the surface expression of CXCR4, thus regulating CXCR4-dependent cell migration [77]. Our integrated data showed that retinal T cells expressed higher levels of Pim1 and Cxcr4 than did T cells from CDLNs, indicating that PIM1 and CXCR4 might synergistically promote T cell ocular infiltration during EAU. As validated by flow cytometry, DMF treatment reduced the expression level of PIM1 and CXCR4 in CD4 + T cells and significantly diminished the ocular infiltrating Teff cells. These results revealed that DMF treatment could suppress ocular infiltration of Teff cells, and this effect might depend on its regulatory effect on PIM1 and CXCR4.

## Conclusions

Overall, in this study, we constructed a post-DMF administration transcriptional atlas and evaluated the effect of DMF on the transcriptomic profiles of diverse immune cell populations during uveitis. Our research indicated that DMF exerts its effect by partly reversing the transcriptional alterations that occur during uveitis. Subsequent studies demonstrated that DMF reversed the imbalance of Teff/Treg through repression of the PIM1-AKT-FOXO1 pathway. In addition, DMF reduced the proportion of PC by inhibiting PIM1 expression. Moreover, DMF also repressed ocular infiltration of Teff cells via inhibition of PIM1 and CXCR4 expression. These findings elucidate the effects of DMF in AU and provide novel insights into the beneficial mechanisms of DMF.

### Electronic supplementary material

Below is the link to the electronic supplementary material.

Supplementary Material 1

Supplementary Material 2

Supplementary Material 3

## Data Availability

The data supporting the conclusions of this article is available in the Genome Sequence Archive, under GSA accession No. CRA014816.

## Abbreviations

ACs: Amacrine cells
AKT: Protein kinase B
AU: Autoimmune uveitis
CBCs: Cone bipolar cells
CDLN: Cervical draining lymph node
cDCs: Conventional dendritic cells
CFA: Complete Freund’s adjuvant
CNS: Central nervous system
Ctl: Cytotoxic T lymphocytes
CONEs: Cone cells
DEG: Differentially expressed gene
DMF: Dimethyl fumarate
EAU: Experimental autoimmune uveitis
FOXO1: Forkhead box O1
GCs: Germinal center B cells
GO: Gene Ontology
IRBP1–20: Interphotoreceptor retinal binding protein 1–20
Macros: Macrophages
MAGs: Macroglia
Monos: Monocytes
Mono&Macro: Monocytes and macrophages
NBCs: Naïve B cells
NCD4: Naïve CD4 + T cells
NCD8: Naïve CD8 + T cells
Neus: Neutrophils
PC: Plasma cells
pDCs: Plasmacytoid dendritic cells
Prot: Proliferative T cells
PTX: Pertussis toxin
RBCs: Rod bipolar cells
RODs: Rod cells
RPEs: Retinal pigmented epithelial cells
scRNA-seq: Single-cell RNA sequencing
Teff: Effector T cells
Tfh: T follicular helper cells
Th1: T helper 1 cells
Th17: T helper 17 cells
Treg: Regulatory T cells
VECs: Vascular endothelial cells
