# Supplementary material for: Beneficial mechanisms of dimethyl fumarate in autoimmune uveitis: insights from single-cell RNA sequencing
Source: J Neuroinflammation. 2024 Apr 29;21:112. doi: 10.1186/s12974-024-03096-6 (PMC11059727; doi:10.1186/s12974-024-03096-6)

**Additional file 1: Figure and Legend**

**Supplementary Figure. 1**

A. Schematic diagram illustrating the experimental design, mouse grouping, modeling time, and dosing schedule.

B. Representative fundus images and clinical scores of EAU mice and EAU mice administered with DMF from day 10 after immunization. Arrows indicate inflammatory exudation and vascular deformation. Each group contained six mice. The values represent the mean ± SD. Significance was determined using Mann-Whitney U test. DMF in established EAU, the group of EAU mice administered with DMF from day 10 after immunization.

C. Heatmap showing the scaled expression of discriminative gene sets for immune cell clusters in CDLNs from all the mouse groups.

D. Volcano plots showing upregulated and downregulated DEGs of all immune cell types in the DMF-treated EAU/Normal comparison groups. The red and blue dots indicate upregulated and downregulated DEGs, respectively.

E. Bar plot showing GO terms enriched by downregulated or upregulated DEGs of all immune cells in the DMF-treated EAU/Normal comparison groups.

F-G. Dot plots showing GO terms enriched with downregulated (F) or upregulated (G) DEGs in the DMF-treated EAU/EAU comparison group in pDCs, macrophages, and neutrophils.

**Supplementary Figure. 2**

A. Heatmap showing the scaled expression of discriminative gene sets for T-cell subsets of CDLNs from all mouse groups.

B-C. Bar plots showing GO terms enriched by upregulated DEGs in the DMF-treated EAU/EAU comparison group in Tfh (B) and Prot (C) cells.

**Supplementary Figure. 3**

A. Representative fundus images and clinical scores of normal and DMF-treated normal mice. Each group contained six mice. The values represent the mean ± SD. Significance was determined using Mann-Whitney U test.

B. Representative fundus hematoxylin and eosin staining plots and pathological scores of normal and DMF-treated normal mice. Scale bars, 20 mm. Each group contained six mice. The values represent the mean ± SD. Significance was determined using Mann-Whitney U test.

C-E. The proportions of Th17 cells (C), Th1 cells (D), and Treg cells (E) among CD4+ T cells from CDLNs of normal mice and DMF-treated normal mice were measured via flow cytometry after immunization on day 14. Each group contained six mice. The data are expressed as the mean ± SD. Significance was determined using unpaired two-tailed Student’s t-test.

**Supplementary Figure. 4**

A. UMAP plots of B-cell subsets from the CDLNs of all the mouse groups.

B. Heatmap showing the scaled expression of discriminative gene sets for B-cell subsets of CDLNs from all mouse groups.

C. UMAP plots of canonical markers for B-cell subsets from all mouse groups.

D. Bar plots showing the percentages of B-cell subsets among CDLNs from normal, EAU, and DMF-treated EAU mice.

E. Pseudotime trajectory analysis of B cells. Cells are colored according to pseudotime (left) or cell type (right).

F. Line plots showing the expression of Pim1 in each B-cell subset.

G. Proportions of Pim1+ cells among CD19+ B cells from CDLNs of EAU mice and DMF-treated EAU mice measured by flow cytometry after immunization on day 14. Each group contained six mice. The data are expressed as the mean ± SD. Significance was determined using unpaired two-tailed Student's t-test.

H. Proportions of PCs measured by flow cytometry after immunization on day 14. Each group contained six mice. The data are expressed as the mean ± SD. Significance was determined using unpaired two-tailed Student's t-test.

I. Line plots showing the expression of Cxcr4 in each B-cell subset.

J. Rose diagram showing the numbers of upregulated and downregulated rescue DEGs in each B-cell subset.

K. Dot plot showing GO terms enriched by downregulated rescue DEGs in NBCs and PCs.

L. Bar plot showing GO terms enriched by downregulated downregulated DEGs in the DMF-treated EAU/EAU group in GCs.

M. Dot plot showing GO terms enriched by upregulated rescue DEGs in NBCs and GCs.

N. Bar plot showing GO terms enriched by upregulated DEGs in the DMF-treated EAU/EAU group in PCs.

**Supplementary Figure. 5**

A. Heatmap showing the scaled expression of discriminative gene sets for retina cells from EAU mice.

B. UMAP plots showing the expression of canonical markers of retina cells from EAU mice.

C. Interaction net count plot of retina cells. The thicker the line represents, the greater the number of interactions between the two cell types.

# Supplementary Figure. 1

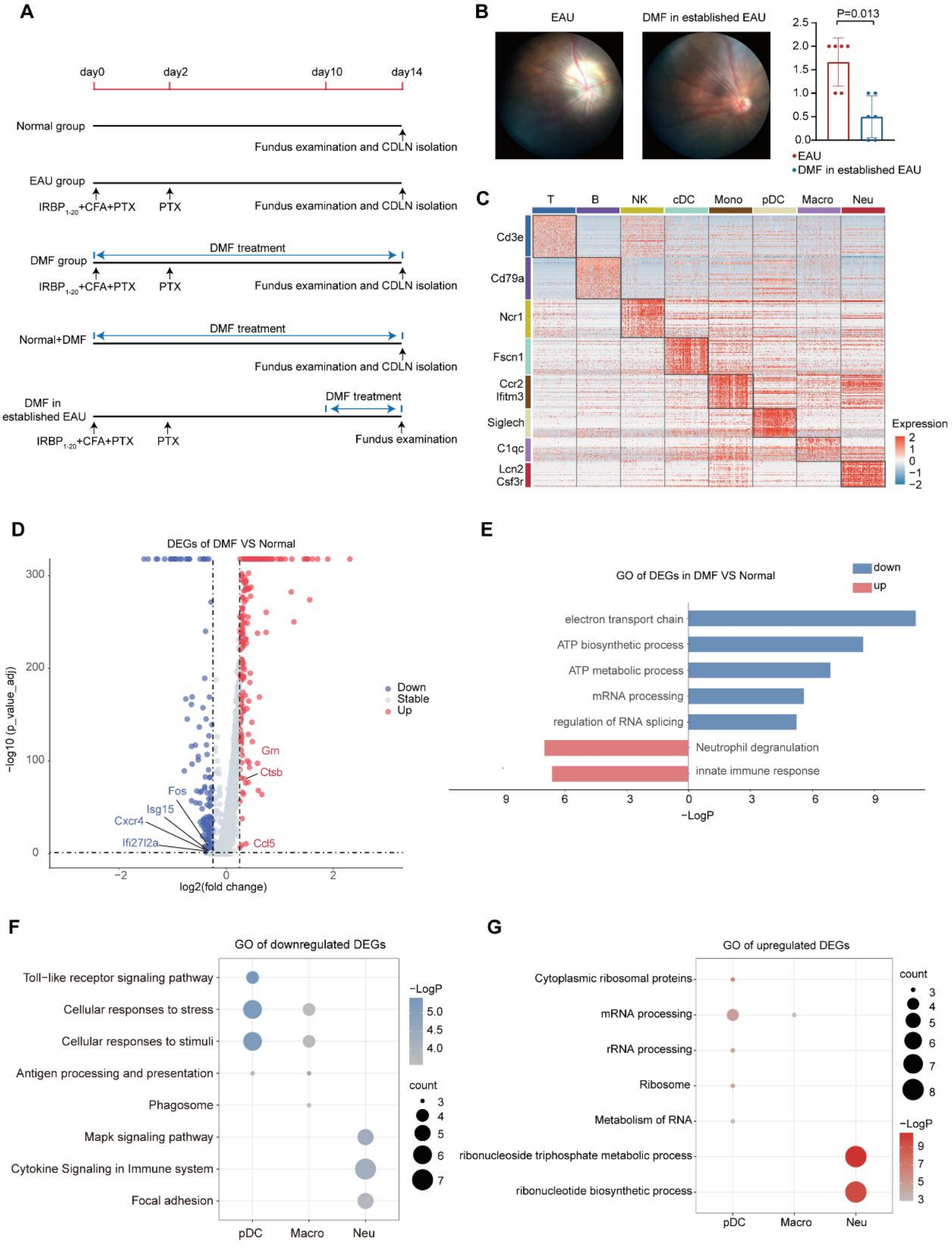

## Supplementary Figure. 2

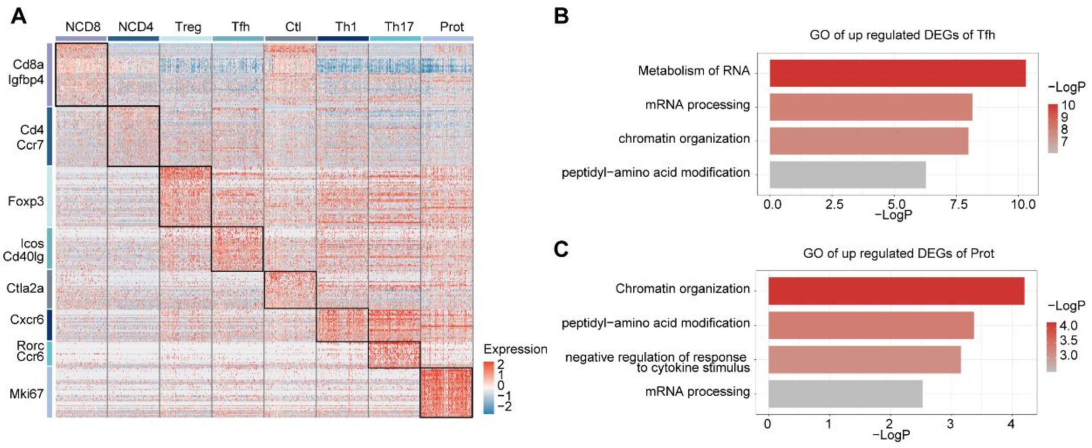

# Supplementary Figure. 3

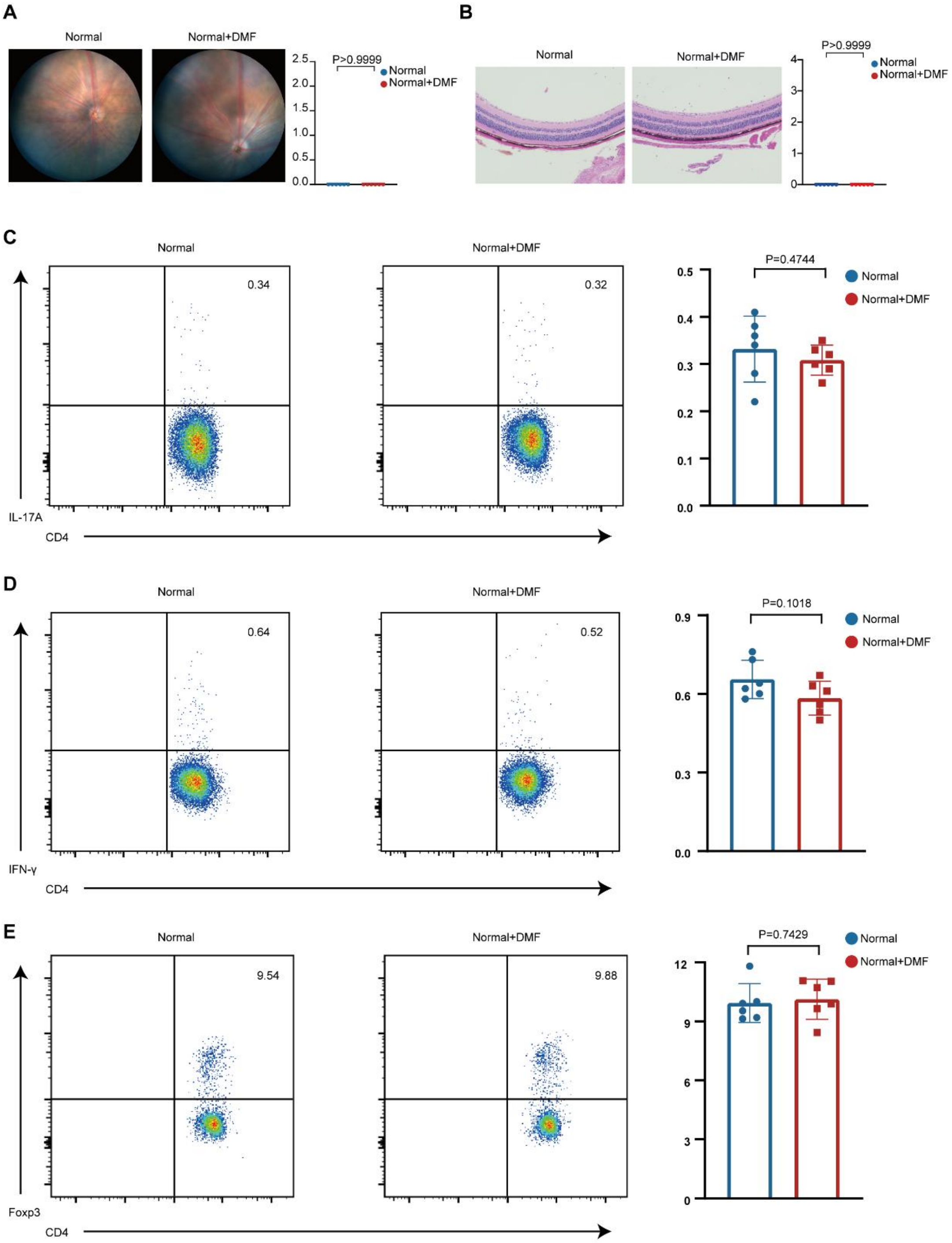

## Supplementary Figure. 4

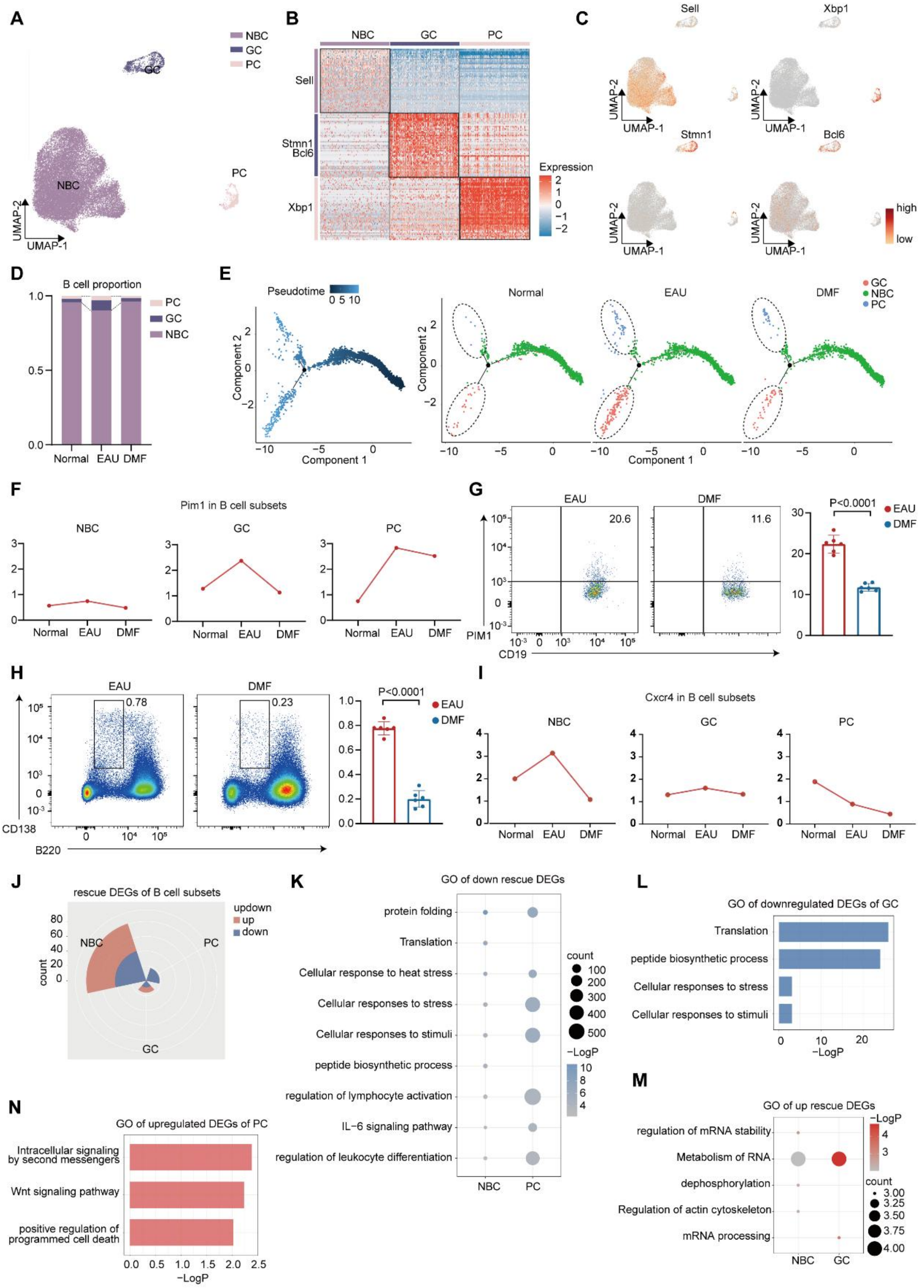

# Supplementary Figure. 5

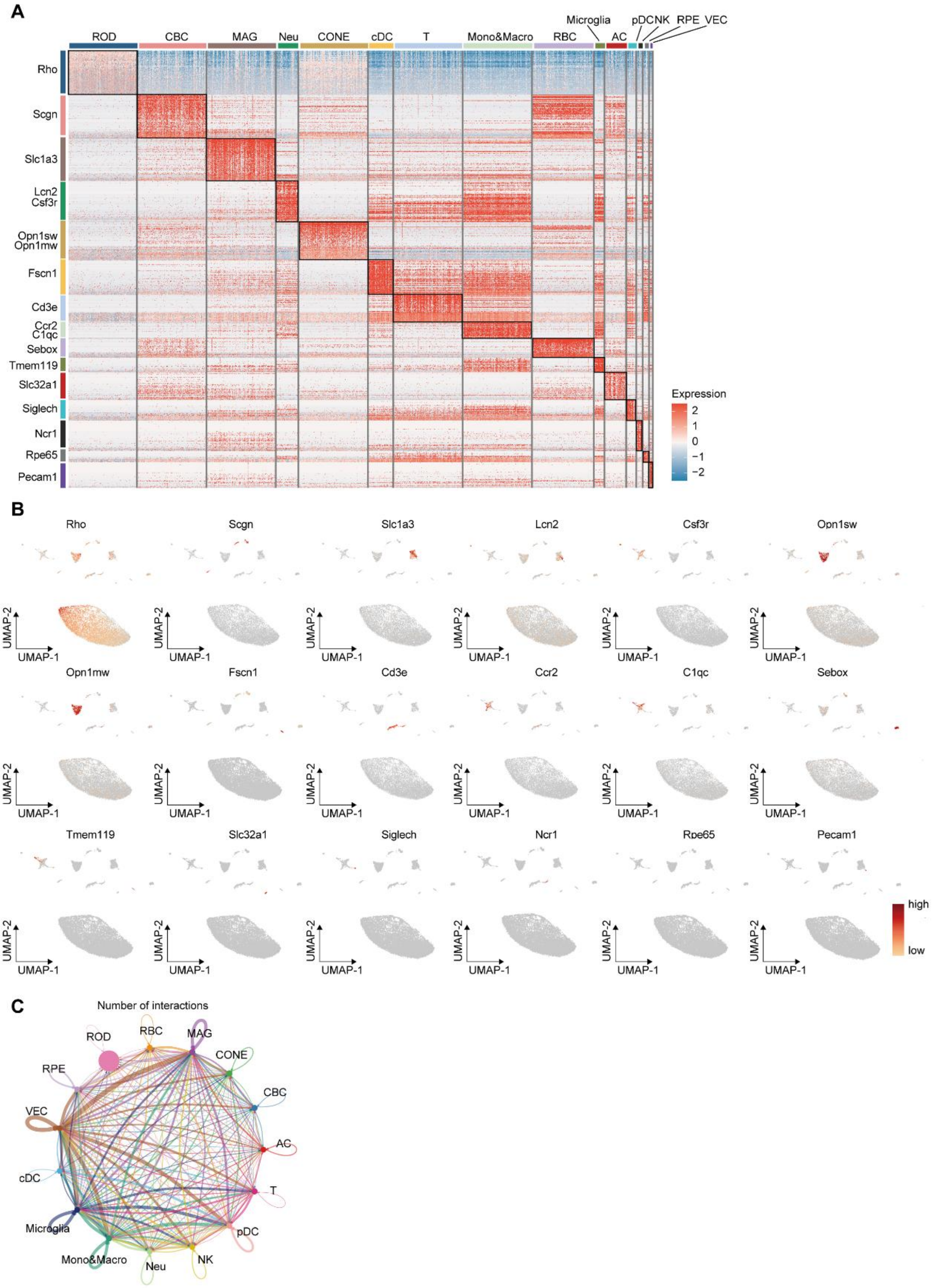

Supplement: Supplementary file 3 — Supplementary Material 3 [file 12974_2024_3096_MOESM3_ESM.pdf]
